# Interaction Mechanisms of Cold Atmospheric Plasmas with HIV Capsid Protein by Reactive Molecular Dynamics Simulation

**DOI:** 10.3390/molecules30010101

**Published:** 2024-12-30

**Authors:** Ying Sun, Yang Chen, Yuantao Zhang

**Affiliations:** School of Electrical Engineering, Shandong University, Jinan 250061, China; ys2018@sdu.edu.cn (Y.S.);

**Keywords:** cold atmospheric plasma, reactive oxygen species, HIV capsid protein, bond breaking and formation, dosage effects

## Abstract

In recent years, plasma medicine has developed rapidly as a new interdisciplinary discipline. However, the key mechanisms of interactions between cold atmospheric plasma (CAP) and biological tissue are still in the exploration stage. In this study, by introducing the reactive molecular dynamics (MD) simulation, the capsid protein (CA) molecule of HIV was selected as the model to investigate the reaction process upon impact by reactive oxygen species (ROS) from CAP and protein molecules at the atomic level. The simulation results show that ground-state oxygen atoms can abstract hydrogen atoms from protein chains and break hydrogen bonds, leading to the destruction of the disulfide bonds, C–C bonds, and C–N bonds. Furthermore, the generation of alcohol-based groups resulting from the impact of ROS can alter the hydrophobicity of molecules and induce damage to the primary, secondary, and tertiary structures of proteins. The dosage effects on the reaction processes and products induced by CAP are also explored with varying numbers of ROS in the simulation box, and the influences on the broken C–H, N–H, and C–N bonds are discussed. In this study, the computational data suggest that severe damage can be caused to CA upon the impact of ROS by revealing the reaction processes and products.

## 1. Introduction

In recent years, with the development of cold atmospheric plasma technology, plasma medicine as an integration of plasma science and biomedical technology has gradually developed [1,2,3]. The experimental and computational results have shown the significant effects of CAP in sterilization and disinfection [4,5], surface modification of materials [6], dental treatment [7], wound healing [8], dermatological treatment [9] and tumor treatment [10,11]. In addition, CAP treatment is also essential for the elimination of human pathogenic viruses. The interactions of CAP and peptides were discussed by experimental measurements and computational data [12,13]. RNA damage by CAP-treated phage protein fragmentation was also investigated [14], and Terrier et al. used CAP to treat influenza A virus to cause virus inactivation [15]. Even the CAP has been applied to interact with cancer cells, and the very positive effects are the new hope for cancer treatment [16,17]. Proteins, biomolecules comprised of amino acids linked by peptide bonds, are ubiquitous in all living organisms. They serve an indispensable function in the viral infection process, constituting the primary structural component of virus particles and participating in crucial aspects such as specific recognition, replication, and release [17]. Consequently, it can be reasonably inferred that the destruction of these proteins has the potential to impede the binding and invasion of viruses into host cells, thereby preventing viral infection. However, a further understanding of the mechanisms of CAP and protein is still limited. Usually, the molecular dynamics (MD) simulation, a powerful tool, is applied to unveil the underpinning interactions [18,19], and the broken and formation of chemical bonds are given in detail based on the simulation results. Recently, with the rapid development of artificial intelligence, deep learning technology has also been introduced to advance the application of plasma medicine [20,21,22], offering new insights into the field of plasma medicine [21].

Human Immunodeficiency Virus (HIV) can cause Acquired Immunodeficiency Syndrome (AIDS). According to the World Health Organization, as of 2017, the number of people infected with HIV worldwide may reach more than 37 million. The virus can destroy the body’s immune system, making the body lose immune function and susceptible to other diseases, with a high fatality rate. Currently, there is still no effective vaccine against the virus and resistance to existing drugs is emerging [23]. The medical community is still actively exploring new ways to cure the virus, and plasma treatment as a new therapeutic method may bring some new progress [24,25,26,27]. In recent years, our group has conducted reactive MD simulations with ReaxFF potential is closely related to plasma medicine applications, with further details available in references [22,25,27,28,29,30,31,32,33,34,35,36,37]. Previous studies by our group have encompassed major cell walls, cell membranes, proteins, fatty acids, antibiotics, and viruses, revealing microscopic reaction mechanisms that agree well with the experimental measurements [18]. Motivated by these findings, it is effective and feasible to explore the microscopic mechanism of damage to the FP by ROS using reactive MD simulation. Additionally, the simulation results serve to complement macroscopic experimental observations, while the experimental outcomes validate the accuracy of our simulations.

In this study, the reactive MD simulation was applied to unveil the reaction processes between ROS, mainly the ground state oxygen atoms, and the capsid protein (CA) of HIV-1 virus with the final products, the bond breaking and formation are explored in detail. The dosage effects of ROS on the bond broken and formation were also calculated by changing the numbers of ROS in the simulation box. Finally, based on the simulation results and data analysis, it is concluded that CAP can effectively disrupt phage proteins by destroying critical chemical bonds.

## 2. Modeling and Simulation

### 2.1. Generation of ROS

The composition of CAP varies greatly depending on the working gas and the discharge parameters [38,39]. It contains various active components, such as electrons, ultraviolet light, and positive and negative ions. The widely accepted theory is that the effect of CAP on biological tissues is mainly through the action of reactive oxygen species (ROS) and reactive nitrogen species (RNS) [40,41]. The air plasma contains a large amount of ROS [42], such as ground and excited state oxygen atoms, OH, $H_{2}O_{2}$, ${\mathrm{HO}}_{2}$, $O_{2}^{-}$, etc., with the active chemical properties, which could be modulated and optimized by discharge parameters [43,44]. In the present study, the ground-state oxygen atoms are selected as the main ROS for the reaction with biological molecules to explore the reaction processes and the final products, discussing the oxidizing ability and processes of ROS.

### 2.2. Description of CA Monomer Model

In 1952, Danish scientist Linderstrom-Lang proposed the concept of the tertiary structure of proteins. The primary structure mainly refers to the sequence of all amino acid residues connected by peptide bonds, including disulfide bonds in protein molecules. The secondary structure refers to the local spatial conformation of the main chain of the protein peptide chain, that is, the relative spatial position of the backbone atom of the peptide chain. Maintaining the stability of the secondary structure mainly depends on the hydrogen bonds inside and between the peptide chains. The tertiary structure refers to the relative spatial position of all amino acid residues in the whole peptide chain, that is, the arrangement position of all the atoms in the whole peptide chain in three-dimensional space. The formation and stability of tertiary structures depend on weak interactions or non-covalent bonds, mainly hydrogen bonds, van der Waals forces, hydrophobicity, etc. Disulfide bonds also play an important role in maintaining conformational stability [45].

**Figure 1:**
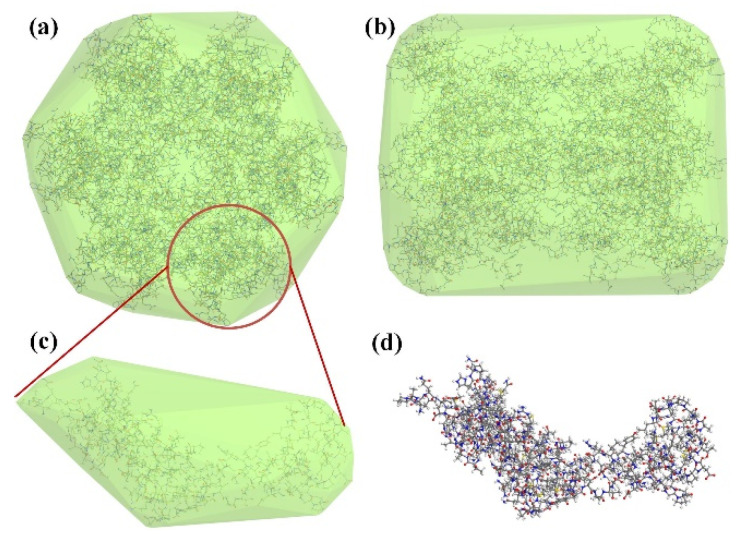
(**a**) Positive view of the capsid protein hexamer structure of HIV-1 virus; (**b**) side view of capsid protein; (**c**) the CA monomer model selected in this study; (**d**) ball and stick model structure of CA monomer.

The capsid protein has a cone-shaped shell structure consisting of 1500 repeating hexamers and a small number of pentamer lattices, in which CA acts as a protective coating for the viral RNA genome and several proteins, including reverse transcriptase and integrase [46]. Figure 1a,b shows the visual images of the hexamer in two directions, respectively. Seen from the front, the hexamer is hexagonal and consists of six symmetrical groups of petal-like protein monomers. From the side view, the hexamer is tubular and divided into two symmetrical parts, each containing six CA monomers. The CA contains two protein subunits, each divided into two domains, the n-terminal (NTD) and the c-terminal (CTD), connected by flexible joints. It is essential to maintain the integrity of the structure and functions of CA, which undertakes different tasks in different periods of viral activity. Only a complete capsid protein that correctly implements the assembly and dissociation process can ensure the effective release, reverse transcription, early synthesis, and other life processes of the HIV genome [46,47,48]. If the CA structure is destroyed or the assembly process is inhibited, the stability of HIV will be affected, resulting in blocked nucleic acid release, weakened viral replication capacity, and even the loss of the ability to infect host cells [49,50]. Even the mutation of some key amino acid residues can lead to a significant decline or loss of the infectivity of CA. Studies have shown that 70% of residues will lead to the loss of infectivity of the virus after mutation, so CA shows extreme genetic vulnerability [50].

When we selected the simulation fragment of CA, we considered its structure to be highly repeatable. In addition, the pentamer and hexamer have very similar tertiary structures, and only slight differences exist in the non-structural domain of capsid protein [51]. Therefore, this paper takes the CA hexamer monomer (PDB ID: 3H47) as the simulation object, which is roughly the part in the red circle in Figure 1a, as shown in Figure 1c. Figure 1d is the ball-and-stick model state of the CA monomer in Figure 1c. CA monomer molecule consists of five elements, C/H/O/N/S, and contains 231 amino acid residues with more than 3000 atoms. Regarding protein structure, the molecule contains 12 amino acid helices of 122 amino acid residues and two double folds of six amino acid residues. Since the CA structure of HIV-1 is highly repetitive, it can be considered that the CA monomer selected in this paper can effectively represent the whole capsid protein molecule to some extent.

### 2.3. Reactive Molecular Dynamics Simulation

The reaction MD with ReaxFF force field is very different from the traditional MD method, which can describe the formation and breaking of chemical bonds, especially considering the non-bond interactions between atoms, then exploring the structural changes of the reactants and revealing the underpinning reaction mechanism. The reaction MD simulation has been applied to investigate the details of biological macromolecules and CAP [52,53,54]. ReaxFF is an empirical field and one of the most widely used parametric reaction fields in plasma medicine. It can describe covalent and ionic bonds [55], and judge whether two atoms can form new bonds or whether the original chemical bonds between atoms still exist by calculating the relationship between bond length, bond level, and bond energy, so as to simulate the occurrence of chemical reactions at the molecular level. When using ReaxFF for calculations, the bond order and polarization charge are calculated for each iteration of the system and counted into the calculation of the system potential energy [56]. In addition, ReaxFF also considers the non-bond interactions of each pair of atoms, including van der Waals forces, Coulomb forces, and hydrogen bonding interactions. In contrast, non-bond interactions such as hydrogen bonding and van der Waals forces are key factors in maintaining the secondary and tertiary structure of proteins. In addition, ReaxFF considers the non-bonding interactions of each pair of atoms, including van der Waals forces, Coulomb forces, and hydrogen bond interactions, which are vital to maintaining the secondary and tertiary structure of proteins. ReaxFF is still under very rapid development and is applied to unveil the mechanisms in plasma medicine [19,57,58].

**Figure 2:**
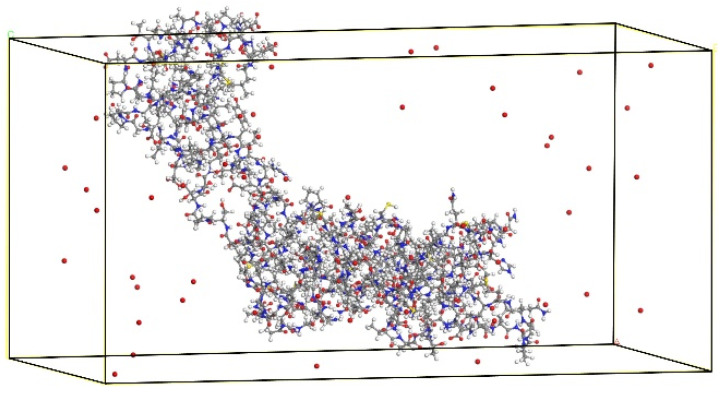
Simulation box with CA molecule and atomic oxygens.

In this study, the CA mono was downloaded from the database PDB band, then imported into the reactive MD simulation, and the optimization process will balance the molecules at room temperature (300 K) under the action of the Berendsen thermostat (reaction relaxation constant is 100 ps). Then, the CA monomer and a number of ground state oxygen atoms were put into a 50 Å × 50 Å × 100 Å simulation box, and the periodic boundary conditions were set to eliminate the influence of the uncertain boundary reaction. The model of the reaction box is shown in Figure 2. The ground state oxygen atoms and monomer structure are randomly distributed in the reaction box. In addition, to distinguish the original oxygen atoms on the CA molecule, we set the diameter of the free oxygen atoms to be slightly larger than that of the CA molecule itself. After optimization, the reaction box was placed in the ReaxFF force field, and the reactive MD simulation was carried out under the NVT canonical ensemble (the model volume was fixed, and energy with the outside world). The system temperature was still set at 300 K, and the equilibrium time was fixed at 50 ps with the reaction time step of 0.1 fs; the total simulation time was ended after 200 ps to ensure all possible reactions were considered with at least 2 million iterations carried out. In the simulation, each calculation was performed at least ten times to obtain the statistical results and for each run of the calculation the reaction sites may be different and the distribution of the final products vary, but the types of bond-breaking and formation are the same, as well as the reaction products. The simulation duration was deemed sufficient to capture the reaction processes leading to bond breaking and formation within the CA structure. Furthermore, the results of the CA reaction exhibited minimal changes upon extending the simulation duration by 50 ps, indicating that 200 ps is adequate for observing the interaction between CA and O atoms.

## 3. Results and Analysis

### 3.1. Bonds Broken and Formation

Based on the computational data, the ground-state oxygen atoms can effectively destroy the microstructure of CA, including the destruction of some chemical bonds on amino acid residues and the generation of hydrophilic groups, which indicates the damage of CA impact by CAP. In this section, the simulation system containing 50 ground-state oxygen atoms is taken as an example to describe in detail the interaction mechanism of ground-state oxygen atoms on various chemical bonds on protein molecules. The simulation results show that the H-abstraction reaction is very crucial for the breaking and formation of chemical bonds in the simulation. Usually, the oxygen atom first captures the hydrogen atom on the side chain of the CA monomer to form the hydroxyl group OH, and this group further takes a hydrogen atom from other positions to form water molecules. At this time, because of the H-abstraction reaction, some atoms are no longer saturated with chemical bonds. In the process of iterative calculation, changes in bond level and polarization charge will lead to the breaking and formation of some other chemical bonds, thus triggering a series of chemical reactions. It is worth noting that the roles of OH radicals from CAP were also explored by reactive MD simulation, because the effects and reaction mechanisms of O atoms and OH radicals are highly consistent in the oxidization of CA protein only very slight differences, and only the simulation data on O reactions are given in the manuscript to show the key reactions and final products.

#### 3.1.1. Destruction of N–H and C–H Bond

**Figure 3:**
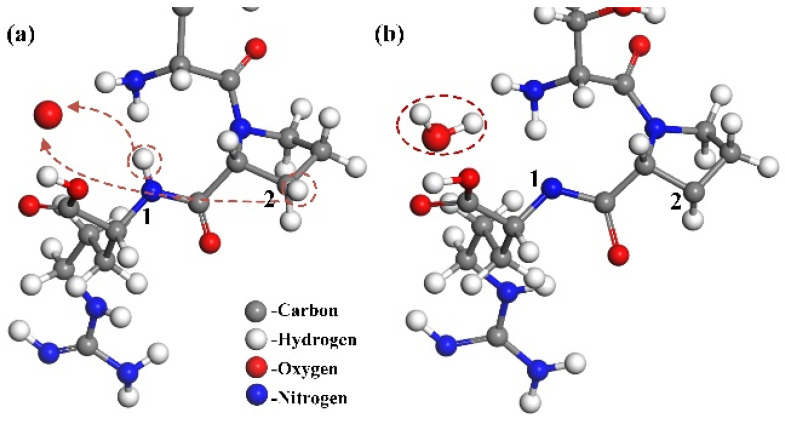
The process of breaking the N–H and C–H bond. (**a**) The oxygen atom interacts with the molecular structure, resulting in the abstraction of H atoms from the $N_{1}$ and $C_{2}$ positions. (**b**) Subsequently, a free H_2_O molecule is formed, leaving the N_1_ and C_2_ positions in an unsaturated state.

According to the simulation results, broken C–H and N–H bonds are the most common reaction types when the ground state oxygen atom interacts with the CA protein. Figure 3 shows the breaking process of C–H and N–H bonds on the no. 17 proline residue and no. 18 arginine residue. The hydrogen atoms attached to the $N_{1}$ and $C_{2}$ atoms were taken away by oxygen atoms to form a water molecule, leaving the bonds of $N_{1}$ and $C_{2}$ atoms unsaturated, but no further reactions between the two atoms took place as the reaction continued. Through dynamic simulation, we can observe that the position of the ground state oxygen atom to seize hydrogen is relatively random, but it mostly happens on the side chain of the protein.

#### 3.1.2. Rupture of Main Chain of CA Monomer

**Figure 4:**
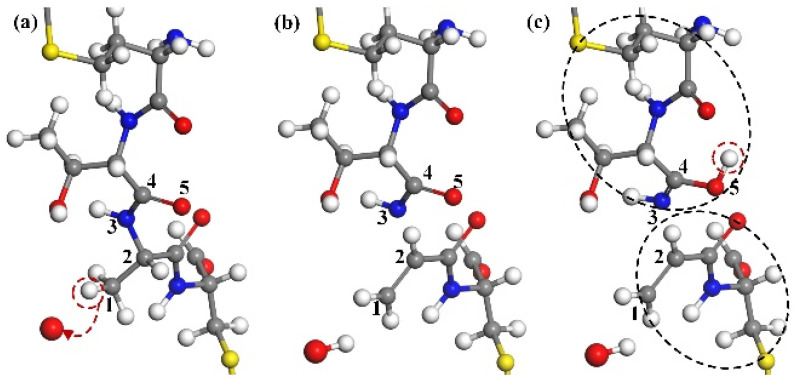
The process of breaking the C–N bond. (**a**) An H atom in the $C_{1}$ position is abstracted by the O atom, leading to the formation of a free hydroxyl radical. (**b**) As a result, the $C_{1}$ site becomes unsaturated, and the $C_{2}$–$N_{3}$ single bond is subsequently cleaved by the interaction with the O atom, yielding a $C_{1}$=$C_{2}$ double bond. (**c**) Ultimately, the structure fragments into two distinct parts.

H-abstraction reaction may have further consequences. In Figure 4, the hydrogen atoms on $C_{1}$ are taken away by oxygen atoms, and the chemical bonds on $C_{1}$ are no longer saturated. There is a tendency for $C_{1}$ and $C_{2}$ to become double bonds, leading to the fracture of $C_{2}$–$N_{3}$ and the formation of $C_{1}$=$C_{2}$. Then, the breaking of $C_{2}$–$N_{3}$ results in the bonds of the $N_{3}$ atom being unsaturated, and forces the $C_{4}$=$O_{5}$ double bond turn to a single one to make the bond of $C_{4}$ accurate. Finally, the $O_{5}$ atom, which has just one bond, obtains a hydrogen atom (red dotted frame on the right) from somewhere else. Thus, the reaction is complete. The destruction of C–N results in the entire structure being separated into the two parts shown in the black circle.

#### 3.1.3. The Production of Alcohol Group

**Figure 5:**
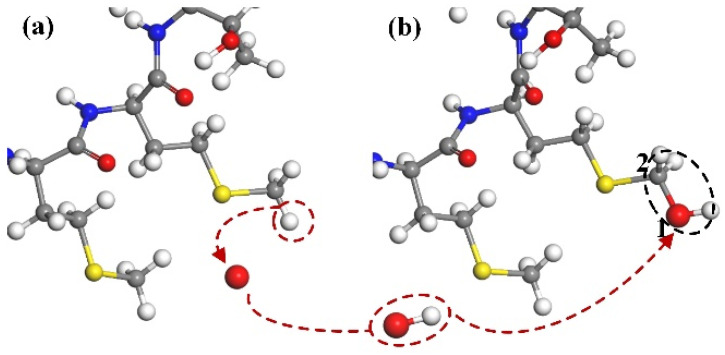
The genration of an alcohol group. (**a**) An H atom in the Ce position is abstracted by the O atom, resulting in the formation of a free hydroxyl radical that is unsaturated at the C position. (**b**) The free hydroxyl radical, possessing a lone electron pair, undergoes adsorption at the C position, ultimately leading to the formation of the alcohol group depicted within the black circle.

In addition to the breaking of chemical bonds, CA monomer molecules also undergo additional reactions, which are also premised on hydrogen capture. In Figure 5, the methyl group ($-{\mathrm{CH}}_{3}$) at the end of the methionine residue is deprived of a hydrogen atom by oxygen to form a hydroxyl group (−OH), which is reattached to the unsaturated $C_{2}$ atom to form a stable alcohol group ($-{\mathrm{CH}}_{2}\mathrm{OH}$). The generation of alcohol-based groups will affect the hydrophilicity of the original CA molecule, thus affecting the spatial conformation of the protein, resulting in changes in the properties of the protein.

#### 3.1.4. The Production of Aldehyde Group

**Figure 6:**
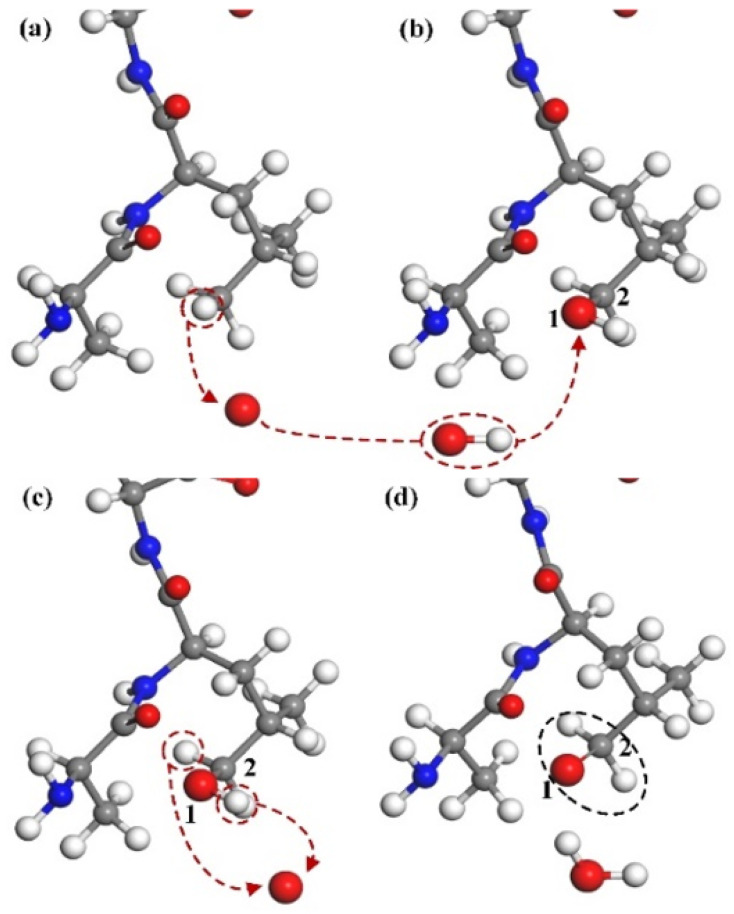
The generation of an aldehyde group. (**a**) An H-abstraction reaction at the C site results in an unsaturated C site. (**b**) Subsequently, adsorption of the free hydroxyl radical at the unsaturated $C_{2}$ position leads to the formation of an alcohol group structure. (**c**) With increasing reaction time and particle concentration, the H atoms at the $C_{2}$ and $O_{1}$ positions undergo further abstraction by oxygen atoms. (**d**) Ultimately, the formation of free water molecules and a $C_{2}$=$O_{1}$ double bond, which constitutes the aldehyde group depicted within the black circle, is observed.

Biomolecules can be oxidized to form alcohol groups, which can be further oxidized to aldehyde groups as oxygen atom concentration continues to increase, as shown in Figure 6. Aldehyde groups are formed based on alcohol groups, and Figure 6a,b shows the process by which leucine residues form alcohol groups. After the formation of the alcohol group, as shown in Figure 6c, the hydrogen atoms on $O_{1}$ and $C_{2}$ on the terminal alcohol group are captured by oxygen atoms to form a water molecule, thus forming a $C_{2}$=$O_{1}$ double bond and eventually an aldehyde group (−CHO) is formed. It is important to note that the aldehyde group appears only when there are a large number of oxygen atoms and is rarely observed at low oxygen concentrations, with only a small number of alcohol groups present.

#### 3.1.5. Destruction of Disulfide Bonds

**Figure 7:**
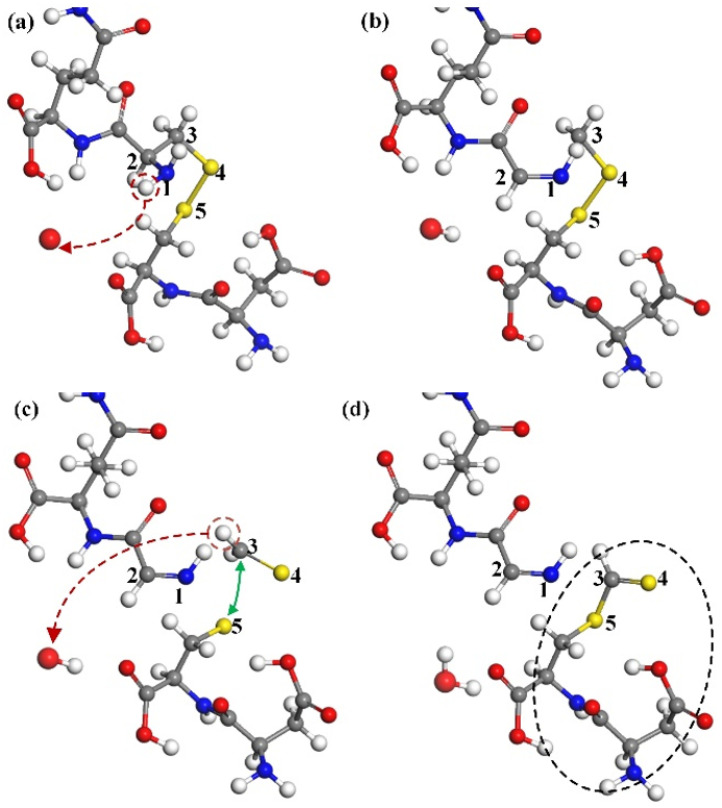
The destruction of disulfide bonds. (**a**) An H-abstraction reaction at the $N_{1}$ position results in the cleavage of the $C_{2}$–$C_{3}$ single bond through impact. (**b**) Consequently, a $C_{2}$=$N_{1}$ double bond is formed. (**c**) The abstraction of an H-atom at the $C_{3}$ position disrupts the disulfide bond. (**d**) Ultimately, a $C_{3}$=$S_{4}$ double bond forms, combining the unsaturated $S_{5}$ and $C_{3}$ sites to establish a new $C_{3}$–$S_{5}$ single bond, resulting in a reconfiguration of bonding and structural change.

The CA monomer protein molecule selected herein also contains a unique disulfide bond, a combination of dehydrogenation of cysteine residues 198 and 218. The disulfide bond belongs to the primary structure of the protein, which stabilizes the spatial structure of the peptide chain and plays an important role in maintaining the stability of the tertiary structure of the protein. After many simulations, we found that the ground state oxygen atom can effectively destroy the disulfide bond, which has many ways to be eliminated. Figure 7 shows a more typical way of destruction.

In Figure 7a, after hydrogen atoms on $N_{1}$ are taken away by oxygen atoms to form hydroxyl, the chemical bond of $N_{1}$ atom is no longer saturated. After the fracture of $C_{2}$–$C_{3}$, $N_{1}$ and $C_{2}$ form a $C_{2}$=$N_{1}$ double bond, as shown in Figure 7b. As the reaction continues, the bond of $C_{3}$ atoms is no longer saturated, forcing the disulfide bond to break, leading to the formation of $C_{3}$=$S_{4}$. In Figure 7c, the hydrogen atoms on $C_{3}$ continue to be captured by the hydroxyl group, so that a single bond of $C_{3}$–$S_{5}$ is formed between the unsaturated $C_{3}$ and $S_{5}$ atoms (shown by the green arrow). Finally, the disulfide bond between the two cysteines breaks, further damaging the primary structure of the CA monomer.

Several of the above reactions promote mutations in amino acid residues, and due to the genetic vulnerability of CA molecules, these large-scale mutations will cause the virus to lose its infectivity [50]. On the other hand, the hydrogen abstraction reaction can change the spatial distribution of the original hydrogen atom and destroy the hydrogen bonding between the main chain of the protein molecule and its side chain. Moreover, the formation of alcohol groups will destroy the hydrophobic action of the original molecule. The destruction of non-covalent bonds, such as hydrogen bonds and hydrophobic interactions, affects the secondary and tertiary structure of proteins [45]. Early experiments with Anfinsen CB demonstrated that the disruption of non-covalent and disulfide bonds, disrupting the secondary and tertiary structure of the molecule, would inactivate the protein [59]. This shows that the ground state oxygen atom can effectively destroy the CA molecule and lose its activity.

Compared with the ordinary H-abstraction reaction, the breaking effect of the C–N and disulfide bonds on the CA monomer is more obvious. Because both structures belong to the category of protein primary structure, which is the basis of protein space conception and specific biological functions [45], the destruction of the primary structure will cause the protein to crack, seriously affecting the assembly ability of the capsid protein, and making the virus infective.

### 3.2. Destruction of the Overall Structure of CA Monomer

**Figure 8:**
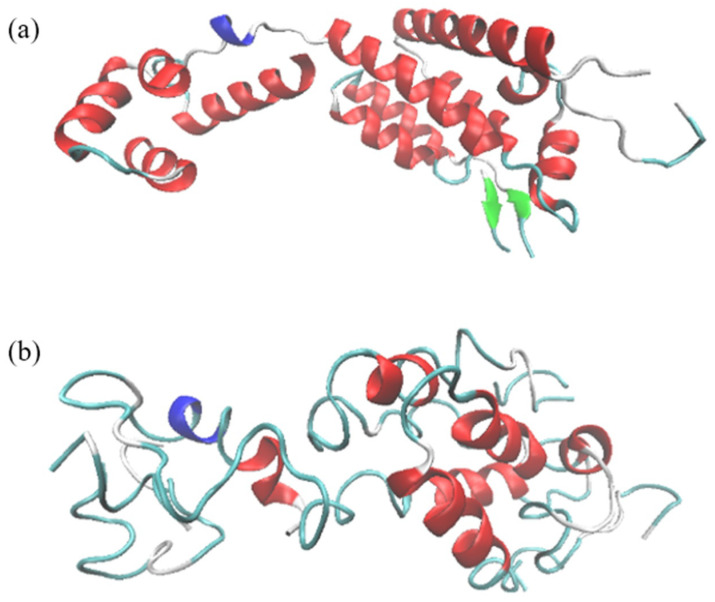
Comparison of structural changes of CA impact by ROS.

The protein secondary structure refers to the local spatial conformation of the main chain constituting the protein peptide chain, that is, the relative spatial position of the main chain skeleton of the peptide chain. It mainly includes α–Helix, β-sheet, Ω-ring, etc., and the stability of maintaining the conformation of these secondary structures depends on the hydrogen bonds inside the peptide chain17. The hydrogen bond is a weak interaction different from the covalent bond. The ReaxFF force field can take into account the non-bond interactions, such as hydrogen bonds, in the calculation. As shown in Figure 8a, the CA monomer is a complete polypeptide chain containing 231 amino acid residues. Its secondary structure consists mainly of 11 α–Helices (red part) and two β-sheets (green arrows) and a 3_10_ spiral (blue part). Figure 8b shows the CA monomer image after the reaction with 50 ground state oxygen atoms. It can be clearly seen from the comparison that only 11 incomplete spiral structures remain in the 11 α–Helices, and the two β-sheet structures disappear, which suggests that the original carbon skeleton of a complete polypeptide chain has been broken into several parts, indicating that the damaging effect of ROS on the CA monomer is pronounced due to the strong oxidizing power.

### 3.3. Dosage Effects

The dosage effects can also be investigated in detail by varying the number of ROS in the simulation box. By considering the number of 10, 20, 30, 40, 50, 100 oxygen atoms in the given simulation box of 50 Å × 50 Å ×100Å, the effects of different concentrations of ROS on the CA monomer can be explored, and the breaking ratios of chemical bonds were analyzed based on the computational data, indicating the breaking ability of ROS for various dosages.

**Figure 9:**
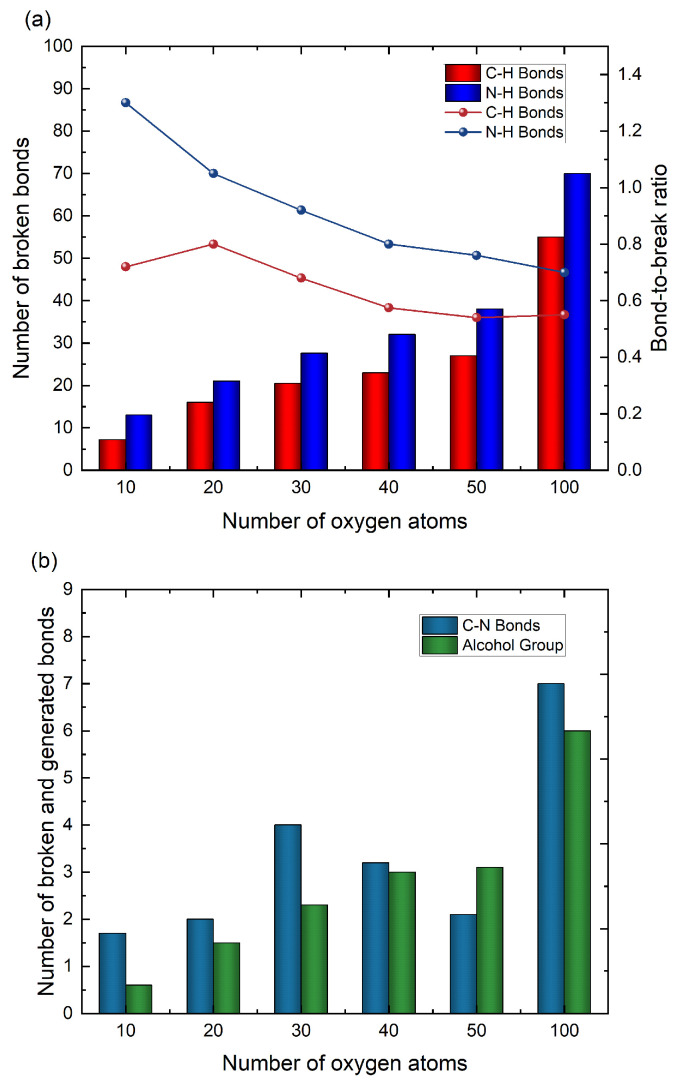
(**a**) The relationship between the number of broken C–H bonds/N–H bonds and the concentration of oxygen atoms. (**b**) The relationship between the number of broken C–N bonds/alcohol groups generated and the concentration of oxygen atoms.

It can be seen from Figure 9a that as the dose of oxygen atoms increases, the number of destroyed C–H and N–H bonds increases. When the number of oxygen atoms is small, the trend of change is almost linear. When the number of oxygen atoms is large enough, the growth rate of the number of broken bonds is gradually reduced, which can also be shown by the physical quantity of the bond-to-break ratio. In this study, the bond-to-break ratio is defined as the ratio of the number of broken bonds and oxygen atoms. It can be seen from the line graph of Figure 9a that as the number of oxygen atoms increases, the bond-to-break ratio decreases. Taking the breaking of the N–H bond as an example, the addition of 10 oxygen atoms can cause an average of 13 N–H bond cleavages, and the bond-to-break ratio is 1.3. After increasing the number of oxygen atoms by an order of magnitude, 100 oxygen atoms can only cause 70 N–H fractures. The bond-to-break ratio is reduced to 0.7, which means that as the number of oxygen atoms increases, the N–H bond becomes more and more difficult to break. On the other hand, in the CA model selected in this paper, the number of C–H bonds is more than 4.1 times the number of N–H bonds. Still, the number of N–H bond cleavages is more than that of C–H bonds, so it can be said that the N–H bond is more easily interrupted by oxygen atoms than the C–H bond.

The computational data show that after the reaction, the polypeptide chain of the CA molecule was no longer complete, and some could be broken into two parts by the impact of ROS. In comparison, others would be broken into three or more parts. Obviously, its breaking was related to the disconnection of the C–N and C–C bonds. As shown in Figure 9b, when oxygen atom concentration is low, there is no obvious linear relationship between the broken C–N bonds and oxygen atoms. The simulation results show that it is still possible to observe the CA molecule break into two parts when only 10 oxygen atoms are applied, which may be caused by the randomness of oxygen atom distribution. The C–H, N–H, or S–H bonds break at random locations where oxygen atoms abstract hydrogen and have a greater impact on the surrounding C–C bonds and C–N bonds, thus contributing to their breakdown. However, when the number of oxygen atoms is increased to 100, it can be visually seen that the amount of C–N bond destruction is still higher than before. The formation of alcohol groups is also affected by the dose level of ROS. It can be seen from Figure 9b that as the number of oxygen atoms increases, the number of alcohol groups formed also increases slowly. The appearance of alcohol groups changes the hydrophilicity of amino acid residues and affects the tertiary structure of molecules.

## 4. Conclusions and Outlook

In this study, the interactions of CAP and the CA of HIV were carefully investigated by reactive MD simulation. From the computational data, ROS can destroy the CA monomer protein molecules by the H-abstraction reaction initially, which is mainly located on the side chain of the protein. When the side chain is stripped of hydrogen atoms, the unsaturated bond atoms will likely continue to set off a chain reaction. On the one hand, the hydrogen capture reaction can change the connection mode between carbon and oxygen/nitrogen atoms, and the simulation also indicates the disulfide and C–N bonds can be destroyed, resulting in the disintegration of macromolecules and severe damage to the primary structure of CA monomer molecules. The α–Helix and β-sheet of CA protein molecules were destroyed by the impact of oxygen atoms in the simulation. The original spatial conformation of protein molecules was significantly changed even without the ability to maintain the original physiological activity, and alcohol and aldehyde groups can change the hydrophilicity of molecules, which is related to the hydrophobicity of proteins. The dosage effects were also discussed based on the simulation results by varying the number of ROS in the simulation box, and the broken C–H/N–H bond is approximately linear with the oxygen atoms in small doses. In this study, the simulation results show that serious damage to CA can be caused by the interaction of CAP, which indicates the potential of CAP to bring new hope for virus treatment.

## Data Availability

The data that support the findings of this study are available from the corresponding author upon reasonable request.
